# Draft Genome Sequence of *Umbilicaria muehlenbergii* KoLRILF000956, a Lichen-Forming Fungus Amenable to Genetic Manipulation

**DOI:** 10.1128/genomeA.00357-14

**Published:** 2014-04-24

**Authors:** Sook-Young Park, Jaeyoung Choi, Gir-Won Lee, Min-Hye Jeong, Jung A Kim, Soon-Ok Oh, Yong-Hwan Lee, Jae-Seoun Hur

**Affiliations:** a Korean Lichen Research Institute, Sunchon National University, Suncheon, South Korea; b Department of Agricultural Biotechnology, Fungal Bioinformatics Laboratory, Center for Fungal Genetic Resources and Center for Fungal Pathogenesis, Seoul National University, Seoul, South Korea

## Abstract

*Umbilicaria muehlenbergii* strain KoLRILF000956 is amenable to *Agrobacterium tumefaciens*-mediated transformation (ATMT), making it the only known genetically tractable lichen-forming fungus to date. We report another advancement in lichen genetics, a draft genome assembly for *U. muehlenbergii* with a size of 34,812,353 bp and a GC content of 47.12%, consisting of seven scaffolds.

## GENOME ANNOUNCEMENT

Over 19% of all known fungal species (approximately 14,000 species) participate in a symbiotic relationship with green algae, cyanobacteria, or both to form lichen. Among them, nearly 98% belong to the phylum *Ascomycota*. The lichens produce useful metabolites with properties of pharmaceutical interest such as anti-HIV, antitumor, antimicrobial, anti-inflammatory, and antioxidant activities. However, unlike other well-studied ascomycetous fungi, including model fungi (1–3) and phytopathogenic fungi (4–7), lichen-forming fungi had no well-established transformation system. Recently, the first successful transformation of a lichen fungus, *Umbilicaria muehlenbergii*, via *Agrobacterium tumefaciens* has been reported (8).

*U. muehlenbergii* belongs to the family *Umbilicariaceae*. The genera in this family are distributed in a wide range of climates, including artic, boreal, austral, temperate, and montane regions (9). *Umbilicaria* spp. contain polysaccharides that showed anti-HIV and antitumor activities (10–12). As *U. muehlenbergii* is dimorphic, it can be grown rapidly in culture in the yeast form, while most other lichen-forming fungi grow extremely slowly in axenic culture (13, 14). This is advantageous in studying lichen biology. Moreover, the recently developed transformation system offers a possibility for systematic gene functional studies. Here, we present the genome sequence of *U. muehlenbergii*, which is anticipated to go hand-in-hand with the aforementioned technological progress to elucidate the genetic blueprint of this fungus.

*Umbilicaria muehlenbergii* strain KoLRILF000956 was isolated from a rock at Mt. Tulaopoding, Jilin Province, China, in 2012. DNA was extracted from the fungus grown in axenic culture using a DNeasy minikit (Qiagen, Valencia, CA), according to the manufacturer’s instructions. The genomic DNA was sequenced at Macrogen, Inc., Seoul, South Korea, by an Illumina HiSeq2000 system using a whole-genome shotgun strategy. The total length of the assembled genome of *U. muehlenbergii* KoLRILF000956 was 34,812,353 bp, with a GC content of 47.12%, representing 663-fold coverage. The genome was assembled into seven scaffolds (≥1,000 bp) using the ALLPATHS-LG assembler (15) and SSPACE version 2.0 (16). Subsequent gene prediction analysis using MAKER (17) yielded a total of 8,294 protein-coding genes. Using the three previously developed gene family pipelines (18–20), we predicted 299 transcription factor genes, 67 cytochrome P450 genes, and 1,488 genes encoding secretory proteins. In addition, 20 putative polyketide synthase genes, containing ketoacyl synthase, acyltransferase, and acyl carrier domains, were predicted by domain search (21).

Since a transformation system has now been developed for *U. muehlenbergii*, the draft genome of *U. muehlenbergii* is a valuable resource for identifying the genes for symbiosis and production of secondary metabolites in lichen, and it also provide a platform to facilitate comparative genomics with other lichen-forming fungi. Moreover, further analysis of the genome will provide insights for functional, biological, and chemical analyses in lichen.

### Nucleotide sequence accession numbers.

The draft genome sequence of *U. muehlenbergii* KoLRILF000956 has been deposited at GenBank under the accession number JFDN00000000. The version described in this article is version JFDN01000000. The scaffold sequences were also deposited at GenBank under accession numbers from KK106981 to KK106987 (seven scaffolds).
